# Flexion‐dependent differences in tibial rotation measured by navigation and robotic systems during bicruciate‐stabilised total knee arthroplasty

**DOI:** 10.1002/jeo2.70749

**Published:** 2026-05-19

**Authors:** Keizo Wada, Daisuke Hamada, Yasuaki Tamaki, Shota Shigekiyo, Yuto Sugimine, Koichi Sairyo

**Affiliations:** ^1^ Department of Orthopedics, Institute of Biomedical Sciences The University of Tokushima Graduate School Tokushima Japan; ^2^ Department of Orthopaedic Surgery Takamatsu Red Cross Hospital Takamatsu Japan

**Keywords:** intraoperative kinematics, navigation system, robotics, total knee arthroplasty

## Abstract

**Purpose:**

This study compared the intraoperative tibial rotational kinematics between navigation‐assisted and robotic‐assisted bicruciate‐stabilised total knee arthroplasty (BCS‐TKA). Although both systems can evaluate knee motion intraoperatively, it is unclear whether they provide comparable rotational measurements. We hypothesised that these systems would show different tibial rotation profiles, particularly in deep knee flexion.

**Methods:**

This retrospective cohort study included 238 knees that underwent BCS‐TKA using an image‐free navigation system (Precision N) or an image‐free handheld robotic‐assisted system (NAVIO or CORI). Tibial rotation relative to the femur was measured intraoperatively through 0° to 120° of knee flexion. Patient‐reported outcomes were assessed using the 2011 Knee Society Score (KSS). Discrepancies in rotational display between the systems were evaluated by a preliminary bench test using a synthetic bone model. Groups were compared using the Kruskal–Wallis test followed by Dunn's post hoc test with Bonferroni correction.

**Results:**

Across all groups, BCS‐TKA demonstrated a characteristic pattern of internal rotation in early flexion, slight external rotation in mid‐flexion and progressive internal rotation towards deep flexion. At 120° of flexion, tibial internal rotation differed significantly among the three groups (*p* < 0.05), with the navigation system displaying greater internal rotation than the robotic system, particularly NAVIO (*p* < 0.01). The robotic system displayed a progressively more externally rotated tibial position beyond 40° of flexion in the bench test. The subjective domains of the KSS improved significantly in all groups at 1 year. Although statistically significant between‐group differences were observed in some domains, these differences were inconsistent, and their clinical relevance remains unclear.

**Conclusions:**

The robotic platform consistently indicated a more externally rotated tibial position compared to the navigation system, suggesting that the two systems apply different reference orientations for tibial rotation in deep knee flexion during BCS‐TKA. Therefore, caution is warranted when comparing intraoperative kinematic data across measurement platforms.

**Level of Evidence:**

Level III, retrospective cohort study.

**Abbreviations**
AP: anteroposterior
BCS: bicruciate‐stabilised
KSS: Knee Society Score
TKA: total knee arthroplasty

## INTRODUCTION

Total knee arthroplasty (TKA) has demonstrated favourable outcomes, but patient satisfaction remains suboptimal. Although multiple factors contribute to patient dissatisfaction, it may be partially explained by changes in knee kinematics after TKA [3]. Bicruciate‐stabilised (BCS) TKA was designed to reproduce more physiological knee kinematics by substituting for both the anterior and posterior cruciate ligaments, thereby aiming to restore native‐like femoral rollback and tibial rotation patterns [7, 17].

For assessing the kinematics of TKA, Banks and Hodge described postoperative TKA kinematics analysis using a combination of fluoroscopic imaging and computer modelling and stated that knee rotations can be measured with an accuracy of approximately 1° and that sagittal plane translations can be measured with an accuracy of approximately 0.5 mm [2]. Using this technique, Dennis et al. demonstrated significant differences between the kinematics of the native knee and those observed after TKA [4]. Meanwhile, with the widespread use of navigation systems, there has been an increasing number of reports on intraoperative kinematic analyses during TKA. Although these assessments rely on manual procedures, previous studies have demonstrated substantial reproducibility and correlation with postoperative kinematic analyses [21, 22]. More recently, intraoperative kinematic patterns have also been linked to postoperative clinical outcomes [20].

With the introduction of robotic‐assisted TKA, more precise bone resection and implant positioning have become possible [14]. Several robotic systems incorporate image‐free technology and register the mechanical axis using methods conceptually similar to those of navigation systems [5, 11]. Therefore, robotic platforms may theoretically provide intraoperative kinematic measurements comparable with those obtained with navigation systems. However, no study has directly compared the intraoperative kinematic measurements obtained by a navigation system and those obtained by a robotic system using the same BCS‐TKA implant.

The purpose of this study was to compare the intraoperative kinematics of the knee between navigation‐assisted and robotic‐assisted TKA using the same implant design. Although both navigation and robotic systems are designed to assess intraoperative knee kinematics, differences in their underlying reference definitions and coordinate systems may result in discrepancies in the measured tibial rotation. Therefore, we hypothesised that the tibial rotation profiles measured by the navigation and robotic systems would differ, potentially reflecting differences in their reference orientations rather than true biomechanical differences.

## MATERIALS AND METHODS

This retrospective cohort study included patients who underwent BCS‐TKA using either an image‐free navigation system (Precision N; Stryker) or an image‐free handheld robotic system (NAVIO or CORI Surgical System; Smith & Nephew) at our institution between September 2017 and July 2024. Inclusion criteria were patients with primary osteoarthritis who underwent primary BCS‐TKA. Exclusion criteria included valgus knees, knee infection, and femoral or tibial fracture. The same prosthesis (JOURNEY II BCS; Smith & Nephew) was used in all patients. The system used changed sequentially over time, with the navigation system primarily used between 2017 and 2019, the NAVIO system between 2020 and 2021, and the CORI system between 2022 and 2024. Therefore, the cohorts represent a historical comparison rather than contemporaneous groups. All procedures were performed by two surgeons, one of whom was a senior surgeon. The senior surgeon was involved in all cases, either as the primary surgeon or as the first assistant, to ensure consistency in surgical technique and intraoperative assessment.

The study was approved by our institutional review board, and informed consent was obtained from all patients for the use of their clinical data for research purposes.

### Surgical technique

All TKAs were performed using a medial parapatellar approach. In group P, navigation‐assisted TKA was performed using the Precision N system. Registration was carried out according to anatomical landmarks. Distal femoral and proximal tibial cuts were made perpendicular to the mechanical axis in the coronal plane. Femoral flexion was targeted at 3°–5°, and the tibial posterior slope was set at 3°. Bone resections were confirmed using the system's verification tool, with additional cuts made as required. Extension and flexion gaps were assessed with implant‐specific spacer blocks, and the rotation and anteroposterior position of the femoral component were adjusted to optimise the flexion gap. Femoral rotation was aligned perpendicular to the surgical epicondylar axis, and tibial rotation was aligned to Akagi's line [1], which was defined intraoperatively by the surgeon based on anatomical landmarks. All implants were cemented, and an inlay‐type patellar component was used in all cases.

In groups N and C, robotic‐assisted TKA was performed using the NAVIO or CORI system. After removal of accessible osteophytes, landmark registration and surface mapping of the distal femur and proximal tibia were performed to construct a three‐dimensional virtual knee model. Neutral mechanical alignment in the coronal plane, femoral flexion of 3°–5°, and a tibial posterior slope of 3° were used as initial parameters. Continuous varus–valgus stress testing throughout the full range of motion was applied to quantify soft‐tissue balance, and the implant position was adjusted to minimise imbalance in the mediolateral gap when necessary. Component orientation was fine‐tuned to within 2° in the coronal plane, with a target global alignment of neutral ±3°. Femoral rotation and anteroposterior positioning were further modified to achieve the optimal flexion gap balance. Tibial rotation was aligned parallel to Akagi's line using the same definition as group P. Distal femoral resection was performed using a high‐speed burr. Proximal tibial resection was performed using the twin‐peg cutting guide under robotic control; when this instrument could not be used because of medial tibial bone loss, a conventional extramedullary guide was used under robotic guidance. In cases where a conventional extramedullary guide was used for tibial resection, the final resection level and alignment were verified using the robotic system. Resections were confirmed, and additional fine‐tuning was performed using a burr when required. Trial components were inserted, and soft‐tissue balance was reassessed. If a residual imbalance remained, the plan was adjusted and reassessed using the robotic platform. After final confirmation, all components were cemented, and an inlay‐type patellar component was implanted in all cases.

### Assessment of intraoperative kinematics

After implantation of all components, the fascia was temporarily closed using two forceps. Kinematics were assessed once in each knee using the navigation system in group P and the robotic system in groups N and C. All knees were flexed manually by the examiner with the heel supported in the open palm of one hand and the knee supported by the other hand. The tourniquet was released during measurement. Care was taken to avoid unintended axial rotation of the tibia throughout the motion.

In group P, the navigation system automatically recorded tibial rotation relative to the femur (positive values indicating internal rotation) at increments of 10° from 0° to 120° of knee flexion. The tibial rotation was calculated based on the relationship between the line perpendicular to the surgical epicondylar axis and Akagi's line, which were defined intraoperatively using anatomical landmarks. The reproducibility of this measurement technique at intervals of 30° has been confirmed previously [22]. The tibial rotation angle was displayed in increments of 0.5°.

In groups N and C, the robotic system automatically displayed tibial rotation relative to the femur (positive for internal rotation) from 0° to 120° of flexion. The robotic system defined the tibial rotation angle in full extension before bone resection as the 0° reference position, and the details of the proprietary computational process underlying this measurement were not available. The tibial rotation angle was displayed in increments of 1°.

### Outcome measures

Patient‐reported outcomes were assessed preoperatively and 1 year postoperatively using the 2011 Knee Society Score (KSS). The subjective component of the KSS includes four domains: Symptoms, Satisfaction, Expectation and Functional activities (walking, standing, and standard, advanced and discretionary activities). The maximum possible score for each subjective domain of the KSS is 25 for Symptoms, 40 for Satisfaction, 15 for Expectation and 100 for Functional activities.

### Preliminary bench test

**Figure 1:**
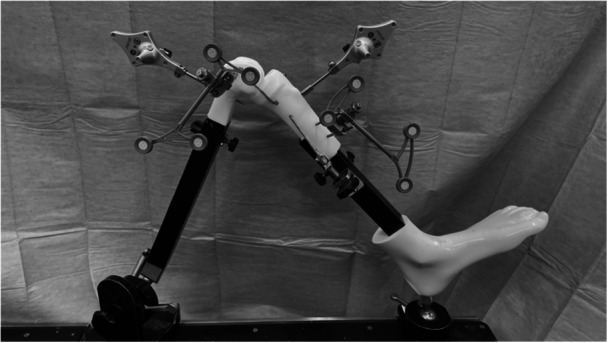
Settings on a synthetic bone model of the knee in a preliminary bench test.

A preliminary bench test was conducted using a synthetic bone model of the knee. The Precision navigation tracker and the NAVIO tracker were mounted simultaneously on the model, and registration was performed for both systems (Figure 1). The tibial rotation angle displayed on the Precision system was manually fixed at 0°. The knee was then flexed in increments of 10° from 0° to 120°, and the tibial rotation angle displayed on the NAVIO system was recorded at each position. For comparison, the tibial rotation angle displayed at full extension (0° flexion) was defined as 0° for both systems. The rotation values recorded by the NAVIO system at each flexion angle were compared with the fixed 0° reference of the Precision system, and the discrepancy between the two systems was documented. This preliminary bench test was designed to qualitatively assess the directional discrepancy between the two systems rather than to provide statistically validated measurements. Therefore, each flexion angle was measured once.

### Statistical analysis

Categorical variables, including sex distribution and operative side, were compared among the three groups using the chi‐squared test. Patient age, KSS subjective domain scores, and tibial internal rotation angles were not normally distributed and were compared among the groups using the Kruskal–Wallis test. When a significant overall difference was detected, post hoc pairwise comparisons were performed using Dunn's test with Bonferroni correction. The Mann–Whitney *U‐*test was used to evaluate differences between preoperative and 1‐year postoperative values for the subjective domains of the KSS within each group.

A post hoc power analysis was conducted using G*Power (version 3.1.9.6; Düsseldorf) based on the observed effect size [6]. The calculated analysis of variance‐equivalent effect size (Cohen's *f*) was 0.17, yielding a statistical power of 0.64 at an alpha level of 0.05. Given this small effect size, an estimated sample size of 339 knees would be required to achieve 80% power for detecting between‐group differences in tibial rotation. All statistical analyses were performed using IBM SPSS Statistics software (version 29.0 for Mac OS X; IBM Corp.). A *p*‐value of <0.05 was considered statistically significant.

## RESULTS

**Figure 2:**
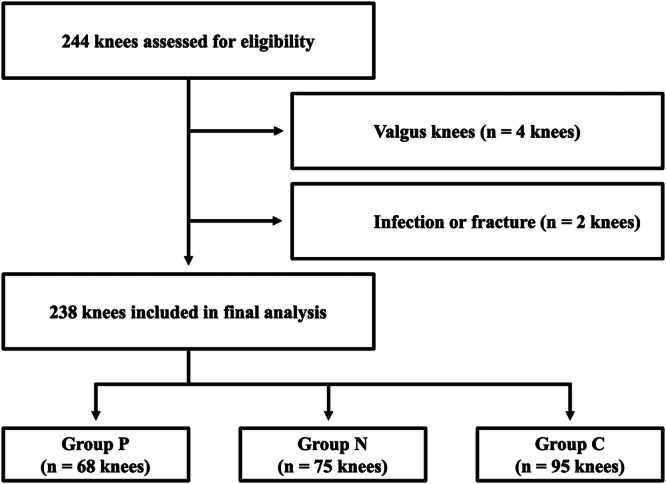
Flow diagram of patient selection. C, CORI‐assisted; N, NAVIO‐assisted; P, precision‐assisted.

A flow diagram of patient selection is shown in Figure 2. After exclusions, 238 knees were included in the final analysis, comprising 68 knees in group P, 75 knees in group N and 95 knees in group C. No significant differences were observed among the groups in baseline characteristics (Table 1).

**Table 1:** Summary of patient demographic data.

	Group P	Group N	Group C	*p*‐value
Knees	68	75	95	
Sex (female/male)	55/13	51/24	74/21	NS
Age (mean)	72.6	72.3	71.1	NS
Laterality (right/left)	35/33	32/43	21/74	NS

Abbreviations: C, CORI‐assisted; N, NAVIO‐assisted; NS, no statistically significant difference; P, precision‐assisted.

### Intraoperative kinematics

**Figure 3:**
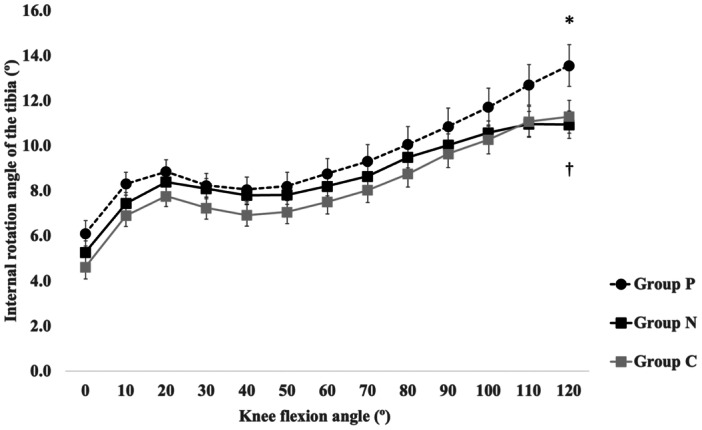
Intraoperative internal rotation of the tibia during knee flexion after bicruciate‐stabilised total knee arthroplasty in the three groups. Error bars indicate the standard error. *Statistically significant difference among the three groups (*p* < 0.05). ^†^Statistically significant difference between group P and group N (*p* < 0.01). N, NAVIO‐assisted; P, precision‐assisted.

Across all three groups, the rotational kinematics of the tibia after BCS‐TKA demonstrated a consistent pattern of internal rotation during early flexion, slight external rotation during mid‐flexion and progressive internal rotation toward deep flexion (Figure 3).

The tibial internal rotation angle differed significantly among the three groups at 120° of flexion (group P: median 14.5° [range −8.5, 27.5]; group N: 10° [−1, 24]; group C: 12° [−9, 26]; Kruskal–Wallis, *H* = 7.39, *p* < 0.025). Post hoc analysis using Dunn's test with Bonferroni correction revealed a significant difference between group P and group N (*p* = 0.008) but no significant difference between group P and group C (*p* = 0.051) or between group N and group C (*p* = 0.383). Overall, internal rotation in deep flexion was greater in group P than in group N or group C.

### Patient‐reported outcomes

KSS subjective domain values improved significantly between the preoperative assessment and the 1‐year postoperative evaluation in all three groups (*p* < 0.001 for all comparisons). Significant differences in the preoperative KSS Expectation and Functional Activities domains were observed among the three groups (*p* < 0.05). At 1 year postoperatively, significant between‐group differences were found in values for the KSS Symptom and Functional Activities domains (*p* < 0.05) (Table 2).

**Table 2:** Values for the subjective domains of the 2011 Knee Society Score before and 1 year after surgery.

		Before surgery		1 year after surgery		
		Median	Range (max, min)	Median	Range (max, min)	*p*‐value
Symptom	Group P	5	23, 0	19*	25, 3	<0.001
	Group N	6	24, 0	22*	25, 5	<0.001
	Group C	6	22, 0	21*	25, 3	<0.001
Satisfaction	Group P	14	30, 0	28	40, 10	<0.001
	Group N	14	38, 0	30	40, 14	<0.001
	Group C	14	32, 0	30	40, 8	<0.001
Expectation	Group P	13*	15, 8	9	15, 3	<0.001
	Group N	14*	15, 9	9	15, 3	<0.001
	Group C	13*	15, 7	9	15, 3	<0.001
Functional activities	Group P	25*	72, 0	58*	93, 5	<0.001
	Group N	31*	78, −6	69*	98, 33	<0.001
	Group C	37*	69, −2	72*	96, 23	<0.001

* *p* < 0.05 for between‐group comparison (Kruskal–Wallis test).

### Preliminary bench test

**Figure 4:**
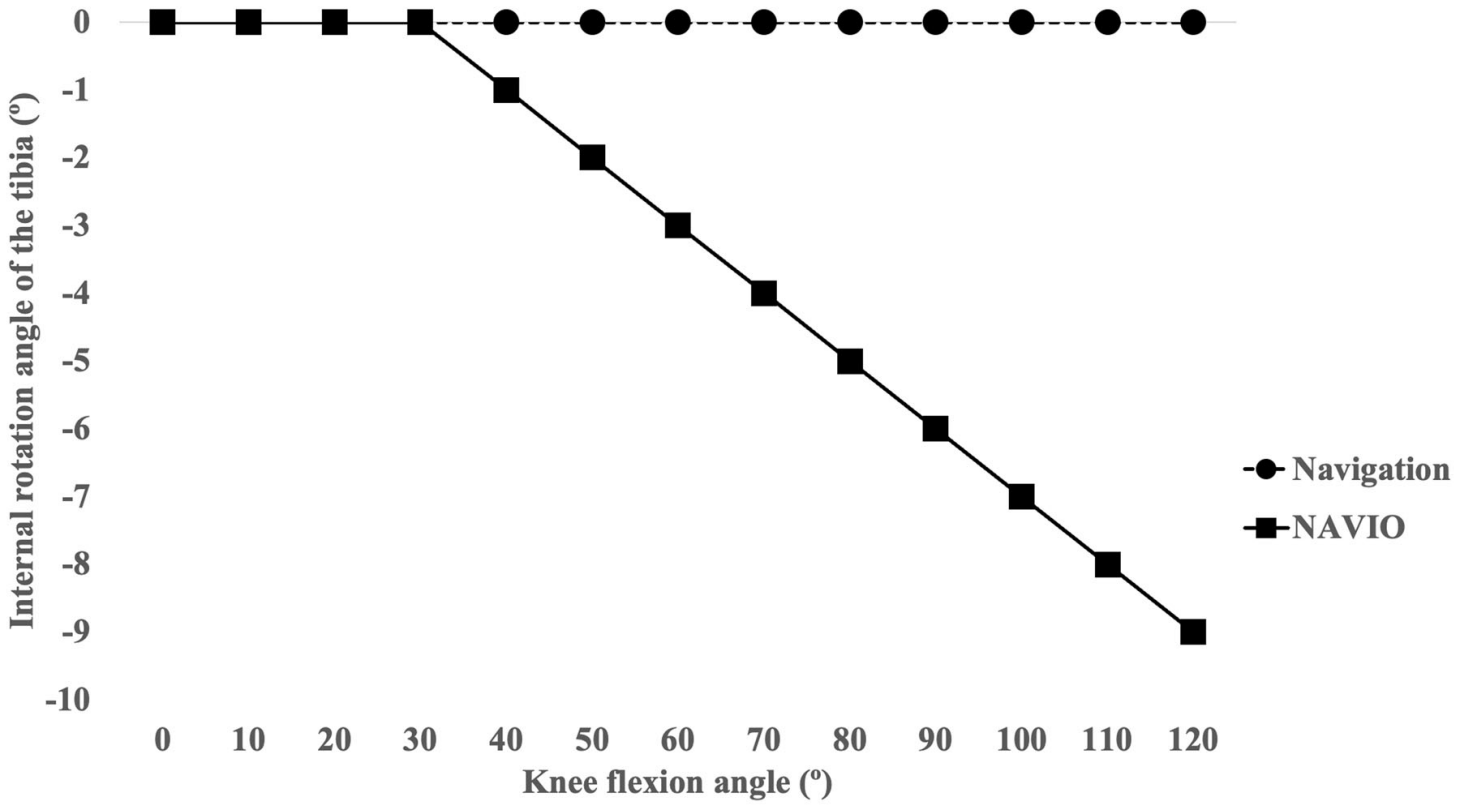
Tibial internal rotation angle during knee flexion recorded by the navigation and NAVIO system in a preliminary bench test. The tibial rotation angle displayed on the navigation system was manually fixed at 0° (black circle with broken line), and the NAVIO system's value (black square with black line) represents the difference in rotational position relative to this reference.

In the synthetic bone model experiment, the tibial rotation angle displayed by the navigation and robotic systems was matched between 0° and 30° of flexion. However, beyond 40° of flexion, the robotic system displayed progressively more external tibial rotation in comparison with the navigation system. The discrepancy increased with knee flexion, reaching approximately 9° at 120° of flexion (Figure 4).

## DISCUSSION

The most important finding of this study was that the tibial internal rotation in deep flexion differed between robotic‐assisted and navigation‐assisted systems, with the robotic system displaying less internal rotation. This discrepancy was consistent with the results of our preliminary bench test, in which the robotic system showed progressively greater external rotation relative to the navigation system when knee flexion exceeded 40°. These findings support our hypothesis that the discrepancies in tibial rotation are primarily attributable to differences in the reference orientation definitions rather than true biomechanical differences. To our knowledge, this is the first study to directly compare intraoperative kinematic measurements between navigation‐assisted and robotic‐assisted TKA using the same implant design.

The observed differences should be interpreted with caution, as they may reflect discrepancies in measurement reference definitions between the systems. In navigation systems, multiple kinematic parameters can be visualised intraoperatively, including axial rotation as well as anterior–posterior and medial–lateral translations [18]. In contrast, robotic systems typically display axial rotation based on a predefined reference position, and the computational process underlying these measurements has not been fully disclosed. These fundamental differences in data acquisition and representation may contribute to the discrepancies observed between the systems. Additionally, the measurement resolution differed between the systems, with the navigation system displaying values in 0.5° increments and the robotic system in 1° increments. While this difference may have influenced the precision of recorded values, it is unlikely to fully account for the magnitude of the discrepancy observed, particularly in deep flexion.

Several studies have shown that BCS‐TKA reproduces some features of the rotational kinematics of the native knee. Grieco et al. [7] compared the in vivo kinematics of normal knees with those of knees that had undergone BCS‐TKA and found that BCS‐TKA restored a kinematic pattern similar to that of the normal knee, although the magnitude of axial rotation differed. Regarding intraoperative assessment, a cadaveric study by Tamaki et al. [17] found that kinematic patterns after BCS‐TKA resembled those of the native knee, despite differences in the initial rotational position of the tibia. Other studies also have demonstrated characteristic patterns of tibial rotation during flexion, including progressive tibial internal rotation [9, 10]. Consistent with these reports, the intraoperative kinematics of the tibia in the present study showed internal rotation in early flexion, slight external rotation in mid‐flexion and gradual internal rotation in deep flexion. In the present results, the tibial rotation angle differed significantly among the three groups at 120° knee flexion. However, patient‐reported outcomes showed no consistent between‐group differences at 1 year. These findings suggest that the observed kinematic discrepancies may have a limited impact on short‐term clinical outcomes and may partly reflect differences in measurement methodology rather than true biomechanical differences.

With the expanding use of robotic‐assisted TKA, several reports have described intraoperative kinematic or soft‐tissue balance assessments obtained from robotic platforms. Previous reports have demonstrated the ability of robotic systems to provide detailed evaluations of gap balance and dynamic alignment throughout the range of motion [5, 12, 16]. Furthermore, Kaneko et al. [11] assessed tibial rotation in bicruciate‐retaining TKA and reported an average intraoperative rotational movement of 12.3°. Although robotic systems provide valuable information about intraoperative kinematics, we found that the tibial rotation angles recorded during robotic‐assisted BCS‐TKA differed from those recorded during navigation‐assisted BCS‐TKA. Importantly, our bench test demonstrated that the robotic system displayed a more externally rotated tibial position than the navigation system beyond 40° of flexion, suggesting that the two systems may use different definitions of the tibial coordinate system or reference point for rotation. Therefore, surgeons should be aware of these system‐specific differences, particularly when interpreting intraoperative kinematic data or comparing findings across platforms. Standardisation of coordinate systems or clearer definitions of the reference position may be necessary to improve comparability.

This study had several limitations. First, it had a retrospective design and did not include data concerning factors that would affect the rotational position of the tibia, such as component alignment [8], soft tissue balance [19] and patient‐specific anatomical factors [13]. Also, the use of different systems over time may have introduced temporal bias. Although it has been reported that the design of the implant plays an important role in the kinematics of TKA [15], further investigation of these factors and their respective confounders is needed. Second, the tibial rotational angle was not assessed under weight‐bearing conditions. Nevertheless, one report suggested that postoperative weight‐bearing kinematics can be predicted by intraoperative non‐weight‐bearing evaluation [21]. Third, we assessed only the tibial rotation angle during flexion. There are no kinematic data available for the anteroposterior, mediolateral and superoinferior dimensions because the kinematics of the knee have six degrees of freedom. In addition, the measurement resolution differed between the systems, which may have influenced the precision recorded. Therefore, further research is needed to clarify the intraoperative kinematics in detail. Fourth, the kinematic analysis was not evaluated for reproducibility. Furthermore, the preliminary bench test was performed only once, which may be problematic because the analysis was performed manually. Nevertheless, a previous study found that intraoperative kinematic analysis was highly reproducible [22]. Therefore, the present data, which were obtained using the same method, are likely to be reproducible. Finally, an a priori sample size calculation was not performed due to the retrospective nature of the study. A post hoc power analysis suggested that the sample size may have been limited, indicating that a larger number of patients would be desirable for more robust statistical power. Despite these limitations, the present study suggests that differences in tibial rotation observed between robotic‐assisted and navigation‐assisted systems may be influenced by differences in measurement reference definitions and methodologies. Surgeons should therefore interpret intraoperative kinematic data with caution, particularly when comparing findings across platforms.

## CONCLUSION

The amount of tibial internal rotation in deep flexion differed between navigation‐assisted and robotic‐assisted BCS‐TKA, with the robotic system displaying less internal rotation. This discrepancy was also observed in a preliminary bench test, suggesting that the two platforms may use different reference orientations for defining tibial rotation. Surgeons should be aware of these differences when interpreting intraoperative kinematic data. Further studies are needed to clarify the underlying coordinate definitions and to determine whether these discrepancies influence long‐term functional outcomes.

## AUTHOR CONTRIBUTIONS

Keizo Wada carried out the kinematic measurements including the preliminary bench test, performed the statistical analysis, and drafted the manuscript. Daisuke Hamada performed the TKA surgery as the surgeon. Yasuaki Tamaki and Shota Shigekiyo assisted in performing the TKA surgery and kinematic measurements. Yuto Sugimine assisted the preliminary bench test. Yuto Sugimine coordinated this study and helped to draft the manuscript. All authors read and approved the final manuscript.

## CONFLICT OF INTEREST STATEMENT

The authors declare no conflicts of interest.

## ETHICS STATEMENT

All performed procedures were approved by the institutional review board of Tokushima University Hospital (the ID number of approval was 2068) and done in accordance with the 1964 Declaration of Helsinki and its later amendments for comparable ethical standards. Informed consent was obtained from all patients.

## Data Availability

The datasets analysed during the current study are not publicly available due to patient privacy considerations but are available from the corresponding author on reasonable request.

## References

[1] Akagi M , Mori S , Nishimura S , Nishimura A , Asano T , Hamanishi C . Variability of extraarticular tibial rotation references for total knee arthroplasty. Clin Orthop Relat Res. 2005;436:172–176. 10.1097/01.blo.0000160027.52481.32 15995437

[2] Banks SA , Hodge WA . Accurate measurement of three‐dimensional knee replacement kinematics using single‐plane fluoroscopy. IEEE Trans Biomed Eng. 1996;43:638–649. 8987268 10.1109/10.495283

[3] Bull AMJ , Kessler O , Alam M , Amis AA . Changes in knee kinematics reflect the articular geometry after arthroplasty. Clin Orthop Relat Res. 2008;466:2491–2499. 18704612 10.1007/s11999-008-0440-z PMC2584306

[4] Dennis DA , Mahfouz MR , Komistek RD , Hoff W . In vivo determination of normal and anterior cruciate ligament‐deficient knee kinematics. J Biomech. 2005;38:241–253. 15598450 10.1016/j.jbiomech.2004.02.042

[5] Faschingbauer M , Freisem K , Khury F , Martin RJ , Bieger R , Reichel H . Tourniquet does not affect intraoperative kinematics during total knee arthroplasty: results of a prospective study using a robotic assistance system. Knee Surg Sports Traumatol Arthrosc. 2024;32:678–684. 38410061 10.1002/ksa.12086

[6] Faul F , Erdfelder E , Lang A‐G , Buchner A . G* Power 3: a flexible statistical power analysis program for the social, behavioral, and biomedical sciences. Behav Res Methods. 2007;39:175–191. 17695343 10.3758/bf03193146

[7] Grieco TF , Sharma A , Dessinger GM , Cates HE , Komistek RD . In vivo kinematic comparison of a bicruciate stabilized total knee arthroplasty and the normal knee using fluoroscopy. J Arthroplast. 2018;33:565–571. 10.1016/j.arth.2017.09.035 29066105

[8] Heyse TJ , Bilal FE‐Z , De Corte R , Chevalier Y , Fuchs‑winkelmann S , Labey L , et al. Internal femoral component malrotation in TKA significantly alters tibiofemoral kinematics. Knee Surg Sports Traumatol Arthrosc. 2018;26(6):1767–1775. 29128876 10.1007/s00167-017-4778-1

[9] Inui H , Taketomi S , Yamagami R , Kono K , Kawaguchi K , Takagi K , et al. Comparison of intraoperative kinematics and their influence on the clinical outcomes between posterior stabilized total knee arthroplasty and bi‐cruciate stabilized total knee arthroplasty. Knee. 2020;27:1263–1270. 32711890 10.1016/j.knee.2020.06.008

[10] Ishibashi T , Tomita T , Yamazaki T , Tsuji S , Yoshikawa H , Sugamoto K . Kinematics of bicruciate and posterior stabilized total knee arthroplasty during deep knee flexion and stair climbing. J Orthop Res. 2021;39:1262–1270. 32510161 10.1002/jor.24773

[11] Kaneko T , Shiga K , Mishima Y . Intraoperative gap assessment in robotic‐assisted bicruciate retaining TKA for knee osteoarthritis. Sci Rep. 2025;15:15675. 40325159 10.1038/s41598-025-99872-2 PMC12052840

[12] Matsumoto T , Nakano N , Hayashi S , Takayama K , Maeda T , Ishida K , et al. Prosthetic orientation, limb alignment, and soft tissue balance with bi‐cruciate stabilized total knee arthroplasty: a comparison between the handheld robot and conventional techniques. Int Orthop. 2023;47:1473–1480. 36928553 10.1007/s00264-023-05737-6 PMC10199853

[13] Nedopil AJ , Delman C , Howell SM , Hull ML . Restoring the patient's pre‐arthritic posterior slope is the correct target for maximizing internal tibial rotation when implanting a PCL retaining TKA with calipered kinematic alignment. J Pers Med. 2021;11:516. 34200031 10.3390/jpm11060516 PMC8228254

[14] Omichi Y , Hamada D , Wada K , Tamaki Y , Shigekiyo S , Sairyo K . Robotic‐assisted total knee arthroplasty improved component alignment in the coronal plane compared with navigation‐assisted total knee arthroplasty: a comparative study. J Robotic Surg. 2023;17:2831–2839. 10.1007/s11701-023-01708-6 37755679

[15] Ong MTY , LaCour MT , Yung PSH , Dessinger GM , Komistek RD . In vivo kinematics for various robotically performed total knee arthroplasty implant designs. J Orthop Res. 2025;43:1284–1292. 40325355 10.1002/jor.26091 PMC12159584

[16] Qordja F , Valpiana P , Andriollo L , Rossi SMP , Salvi AG , Bocchino G , et al. The HKA axis varies significantly with knee motion: a robot‐assisted intraoperative evaluation during total knee arthroplasty supports the use of dynamic, not static, alignment classifications. J Exp Orthop. 2025;12:e70370. 40689101 10.1002/jeo2.70370 PMC12272511

[17] Tamaki Y , Hamada D , Wada K , Takasago T , Nitta A , Omichi Y , et al. Kinematic comparison between the knee after bicruciate stabilized total knee arthroplasty and the native knee: a cadaveric study. Knee. 2023;42:289–296. 37120864 10.1016/j.knee.2023.04.004

[18] Wada K , Hamada D , Takasago T , Kamada M , Goto T , Tsuruo Y , et al. Intraoperative analysis of the kinematics of the native knee including two‐dimensional translation of the femur using a navigation system: a cadaveric study. J Med Invest. 2019;66:367–371. 31656308 10.2152/jmi.66.367

[19] Wada K , Hamada D , Tamaki S , Higashino K , Fukui Y , Sairyo K . Influence of medial collateral ligament release for internal rotation of tibia in posterior‐stabilized total knee arthroplasty: a cadaveric study. J Arthroplast. 2017;32:270–273. 10.1016/j.arth.2016.06.013 27460300

[20] Wada K , Hamada D , Tamaki Y , Shigekiyo S , Sairyo K . Relationship between intraoperative knee kinematics assessed by navigation system and patient‐reported outcomes of bicruciate‐stabilized total knee arthroplasty. Knee. 2025;57:438–443. 41135283 10.1016/j.knee.2025.09.012

[21] Wada K , Mikami H , Hamada D , Yamazaki T , Tomita T , Sairyo K . Can intraoperative kinematic analysis predict postoperative kinematics following total knee arthroplasty? A preliminary. J Med Invest. 2017;65:21–26. 29593171 10.2152/jmi.65.21

[22] Wada K , Mikami H , Hamada D , Yonezu H , Oba K , Sairyo K . Measurement of rotational and coronal alignment in total knee arthroplasty using a navigation system is reproducible. Arch Orthop Trauma Surg. 2016;136:271–276. 26739138 10.1007/s00402-015-2402-8

